# Determinants of automatic age and race bias: ingroup-outgroup distinction salience moderates automatic evaluations of social groups

**DOI:** 10.3389/fpsyg.2024.1328775

**Published:** 2024-03-18

**Authors:** Stephanie Heitmann, Regina Reichardt

**Affiliations:** ^1^Schumpeter Center for Research on Socio-Economic Change, Friedrich Schiller University, Jena, Germany; ^2^Department of Psychology, University of Regensburg, Regensburg, Germany

**Keywords:** ingroup-outgroup salience, social categorization, intergroup bias, age bias, race bias

## Abstract

**Introduction:**

The present research investigates whether ingroup-outgroup distinction salience moderates automatic intergroup bias (i.e., more positive evaluations of ingroup targets relative to outgroup targets) toward multiply categorizable social targets.

**Methods:**

In two experiments, we manipulated the salience of participants’ social identity based on age vs. race, respectively. Afterwards, we measured automatic evaluations of social targets varying in age and race.

**Results:**

Young White participants exhibited higher automatic race bias when their racial identity (i.e., White in contrast to Black) was salient. Conversely, they exhibited higher automatic age bias when their age identity (i.e., young in contrast to old) was salient.

**Discussion:**

Going beyond previous research, we show that it is sufficient to direct participants’ attention to their ingroup-identity in contrast to the respective outgroup to cause changes in automatic intergroup bias. This is important because it provides a strong test of the hypothesis that ingroup-outgroup distinction salience moderates automatic intergroup bias.

## 1 Introduction

To handle the wealth of social information with which people are confronted, they categorize individuals into social groups. Categorizing oneself and others into social groups leads to a distinction between ingroup and outgroup, resulting in intergroup bias, i.e., the phenomenon that ingroup members are evaluated more positively than outgroup members (Tajfel, 1982; Hewstone et al., 2002). Although virtually any category can serve as a basis for social categorization, individuals use primary categories such as age and race most readily because those are perceptually salient and processed automatically (Brewer and Lui, 1989; Ito and Urland, 2005; but see Connor et al., 2023). As a consequence, individuals typically exhibit a strong automatic intergroup bias toward members of the social categories age and race (Nosek et al., 2007).

As age and race bias can have severe detrimental consequences to individuals and societies, it is of utmost importance to understand their determinants. The goal of the present research was to extend our knowledge on moderating factors. To this end, we focused on ingroup-outgroup salience in the context of multiply categorizable social targets. As individuals belong to multiple social categories, the ingroup-outgroup status of social targets depends on which category is used to distinguish ingroup from outgroup (Crisp and Hewstone, 2007). For instance, if a young White person focuses on her group membership as White, then any White person, regardless of their age, may be perceived as an ingroup member while any Black person, regardless of their age, may be perceived as an outgroup member. Conversely, if a young White person focuses on her group membership as young, then any young person, regardless of their race, may be perceived as an ingroup member while any old person, regardless of their race, may be perceived as an outgroup member. As a consequence, automatic age and race bias may change accordingly. In essence, manipulating which particular ingroup-outgroup distinction is salient in the context of multiply categorizable social targets should moderate automatic intergroup bias.

In a related vein, previous research showed that social categorization moderates automatic social evaluation (Mitchell et al., 2003; Gawronski et al., 2010; Jones and Fazio, 2010; Todd et al., 2021). However, these studies differ in important ways from our studies. Most of these studies directly manipulated the categorization of the social stimuli that were presented during the automatic bias measure. For instance, Mitchell et al. (2003) manipulated whether participants categorized the exemplars in an Implicit Association Test based on race or another social category. In other studies (Gawronski et al., 2010; Jones and Fazio, 2010), participants were asked to count how many Black and White faces (vs. young and old faces) appeared as primes during an Evaluative Priming Task. To accomplish this, participants needed to categorize the prime faces according to one dimension (e.g., race), while ignoring the other dimension. In some studies, categorization during the automatic evaluation measure was manipulated indirectly by varying distractor items (Mitchell et al., 2003) or by blocked presentation (Todd et al., 2021). Although more indirectly, these manipulations nevertheless targeted the categorization of the social stimuli shown during the automatic evaluation measure. In several other studies, Todd et al. (2021) administered a classification task before participants completed the automatic bias measure. In this classification task, participants were presented with the faces that were later shown during the automatic bias measure and were asked to classify them according to race or another dimension. This manipulation involved practicing a categorization rule which participants may later apply to the faces in the automatic bias measure as well. Although in one study novel faces were shown in the automatic bias measure, the categorization task may nevertheless have a lasting effect on categorization processes in the automatic bias measure via procedural priming (Smith and Branscombe, 1987, 1988). In essence, all manipulations were directed at the process of categorizing the social stimuli toward which automatic evaluation was being measured. It remains an open question whether this process of categorizing is necessary for the effects to occur.

Other scientists have manipulated the ingroup-outgroup status of social targets by employing a minimal group paradigm (Van Bavel and Cunningham, 2009). White participants were assigned to one of two fictitious teams that were mixed in race. During a learning phase, participants repeatedly categorized photos of White and Black people according to their team membership. An Evaluative Priming Task administered afterwards, revealed that participants automatically evaluated ingroup members more positively than outgroup members, regardless of their race. Again, these studies involved practicing a particular categorization rule with respect to the social exemplars toward which automatic evaluations were assessed. Furthermore, assigning participants to one of two teams may have created a context of cooperation within the own team, which in turn may have enhanced positive evaluations of the ingroup members (Xiao and Van Bavel, 2019). Finally, the learning phase of classifying the photos into the teams may have induced positive attitudes toward the ingroup members and negative attitudes toward the outgroup members via evaluative learning mechanisms (De Houwer et al., 2001; Castelli et al., 2004).

Going beyond previous research, we sought to manipulate ingroup-outgroup distinction salience not by manipulating the categorization of the social exemplars toward which automatic evaluation was being measured, but by simply directing participants’ attention to their own ingroup-identity and the respective outgroup. We sought to measure automatic evaluation of novel multiply categorizable targets that were completely irrelevant to our manipulation of ingroup-outgroup distinction salience. In contrast to previous studies, our manipulation did neither involve a context of cooperation nor a previous learning phase regarding group membership. If we find that such a manipulation is sufficient to change automatic intergroup bias, this would be strong evidence for the hypothesis that mere ingroup-outgroup distinction salience moderates automatic intergroup bias.

To investigate whether the salience of an ingroup-outgroup distinction moderates automatic intergroup bias, we adapted manipulations from social identity research that do not rest on categorizing social exemplars (Haslam et al., 1992, 1999). In Experiment 1, we asked White young participants to describe how they, as a White (vs. young) person, differed from Black (vs. old) people. They then completed an Evaluative Priming Task (EPT; Fazio et al., 1986), measuring automatic age and race bias. Experiment 2 was designed to further test the necessary conditions under which changes in automatic intergroup bias would occur. To this end, we replicated the manipulation of Experiment 1 and included a second condition, that used a more subtle manipulation of ingroup-outgroup distinction salience. In this condition, we told participants that the data of White and Black (vs. young and old) participants would be compared and asked them to indicate their group membership with respect to race (vs. age). We predicted that the manipulations of ingroup-outgroup distinction salience would moderate automatic intergroup bias. Automatic race bias should be higher when White-Black was salient as compared to when young-old was salient. Automatic age bias should be higher when young-old was salient as compared to when White-Black was salient.

Materials and data of all experiments reported in this article are available at osf.^1^ We report all manipulations, measures, and exclusions. The experiments were programmed in DirectRT and MediaLab (Empirisoft Corporation, 2011a,b).

## 2 Experiment 1

### 2.1 Method

Adapting a manipulation from social identity research (Haslam et al., 1999), we asked young White participants to describe how they, as a White (vs. young) person, differed from Black (vs. old) people. To measure automatic race and age bias, participants then completed an EPT (Fazio et al., 1986), showing photos of faces varying in race and age as primes. The design was a 2 (ingroup-outgroup salience: White-Black vs. young-old) × 2 (evaluation score: race bias vs. age bias) mixed design with the latter variable manipulated within-subjects.

#### 2.1.1 Participants

Participants were 74 females recruited from the pool of first-year psychology students and from the department’s study participation system (non-psychology students) at a German University. We recruited only females because this experiment was the first of a battery and the subsequent experiment was restricted to female participants. Psychology students received partial course credit; others received €6 as compensation. As our manipulation was developed for young White people, we excluded participants who were non-White (none) or aged 40 years or older (two participants). The age cutoff was based on aging research, in which people up to 39 years are typically categorized as young adults (MacPherson et al., 2002; Borod et al., 2004). One participant was excluded because part of her data was lost due to technical problems. The age of the remaining 71 participants ranged from 19 to 38 years (*M* = 23.90, *SD* = 3.95). We determined the sample size based on previous studies (Van Bavel and Cunningham, 2009). A sensitivity power analysis, conducted with G*Power (Faul et al., 2007), revealed that with a sample size of 71 and a correlation among the measures of *r* = 0.08, the minimum effect size of a between-within interaction effect we could detect with 80% power was *f* = 0.23. Given the observed effect size of η*_p_*^2^ = 0.14 (*f* = 0.41), power was sufficient.

#### 2.1.2 Procedure and materials

##### 2.1.2.1 Manipulation of ingroup-outgroup distinction salience

After reading general instructions, half of the participants were asked to describe how they, as a White person, differed from Black people. The other half of participants were asked to describe how they, as a young person, differed from old people (Haslam et al., 1999). Participants were asked to write three to five sentences.

##### 2.1.2.2 Evaluative priming task (EPT)

Immediately after the manipulation, participants completed an EPT (Fazio et al., 1986). As prime stimuli, we used a total of 40 head-and-shoulder color photographs of female persons, 10 for each prime category (young White, old White, young Black, old Black), selected from the CAL/PAL Database (Minear and Park, 2004). We used only female persons to match participant gender. As target words, we used 10 positive and 10 negative nouns (Klauer and Musch, 1999). Participants first completed 20 practice trials with targets only. Then, they completed 160 test trials. Each prime picture was presented four times and was paired equally often with a positive and a negative target word. Each target word was presented eight times. All trials were presented in random order. Each trial began with the presentation of a fixation cross for 500 ms, followed by a prime for 200 ms, followed by a target word until participants responded. Participants had to indicate the valence of the target word as quickly as possible, by pressing the ‘I’-key with the right index finger for positive words and the ‘E’-key with the left index finger for negative words. The inter-trial interval was 500 ms. In case participants’ response time was slower than 1,500 ms, the message that they responded too slowly and should respond faster appeared for 1,500 ms after the response. Incorrect responses were followed by an error message for 500 ms.

##### 2.1.2.3 Social identification measure

To explore the mechanisms underlying the potential effects of ingroup-outgroup salience on automatic evaluation, we included a measure of social identification. To assess participants’ degree of social identification with White or young people, participants completed two paper and pencil versions of the Inclusion of Ingroup in the Self Measure (IIS; Tropp and Wright, 2001). The IIS shows seven pairs of circles that differ in their distance and degree of overlap, respectively. The left circle was labeled *me*, the right circle was labeled *young people* on one version, and *White people* on the other version. Participants indicated which pair of circles best described their degree of identification with White or young people, respectively. Responses were coded from 1 *(no overlap)* to 7 *(complete overlap)*.

##### 2.1.2.4 Self-reported evaluations

After completion of the IIS, self-reported evaluations of the 40 target pictures of White young, White old, Black young, and Black old people were assessed using feeling thermometers. Participants indicated how warm their feelings toward each target were on a 7-point Likert scale ranging from 1 *(very cold)* to 7 *(very warm)*. All 40 trials were presented in random order. Finally, participants answered demographic questions, were thanked and debriefed.

### 2.2 Results

The data were analyzed using IBM SPSS Statistics. Confidence intervals were computed using the script CI-R2-SPSS.^2^ For partial eta squared effect sizes, 90% CIs were used.^3^

#### 2.2.1 Automatic evaluation

Following the recommendations of Koppehele-Gossel et al. (2020), we discarded incorrect responses (4.5%) and responses that were faster than 150 ms (1 response) or slower than 1,000 ms (1.7%). Following the recommendations of Ratcliff (1993), we validated the results by applying two alternative procedures of outlier corrections. A cutoff at 1,250 ms (Gawronski et al., 2010) as well as log-transformation of response latencies after applying a cutoff at 1,500 ms yielded the same pattern of results in Experiments 1 and 2. Means and standard deviations of the response latencies are presented in the Supplementary Table S1. To test our hypotheses, we followed the data analyses procedure by Gawronski et al. (2010) and calculated automatic race bias scores and automatic age bias scores. To calculate automatic race bias scores, we subtracted the mean response latency to positive words preceded by a White prime from the mean response latency to positive words preceded by a Black prime, as well as the mean response latency to negative words preceded by a Black prime from the mean response latency to negative words preceded by a White prime. The resulting difference scores were then averaged as an index of automatic race bias. To calculate automatic age bias scores, we subtracted the mean response latency to positive words preceded by a young prime from the mean response latency to positive words preceded by an old prime, as well as the mean response latency to negative words preceded by an old prime from the mean response latency to negative words preceded by a young prime. The resulting difference scores were then averaged as an index of automatic age bias.

**Figure 1:**
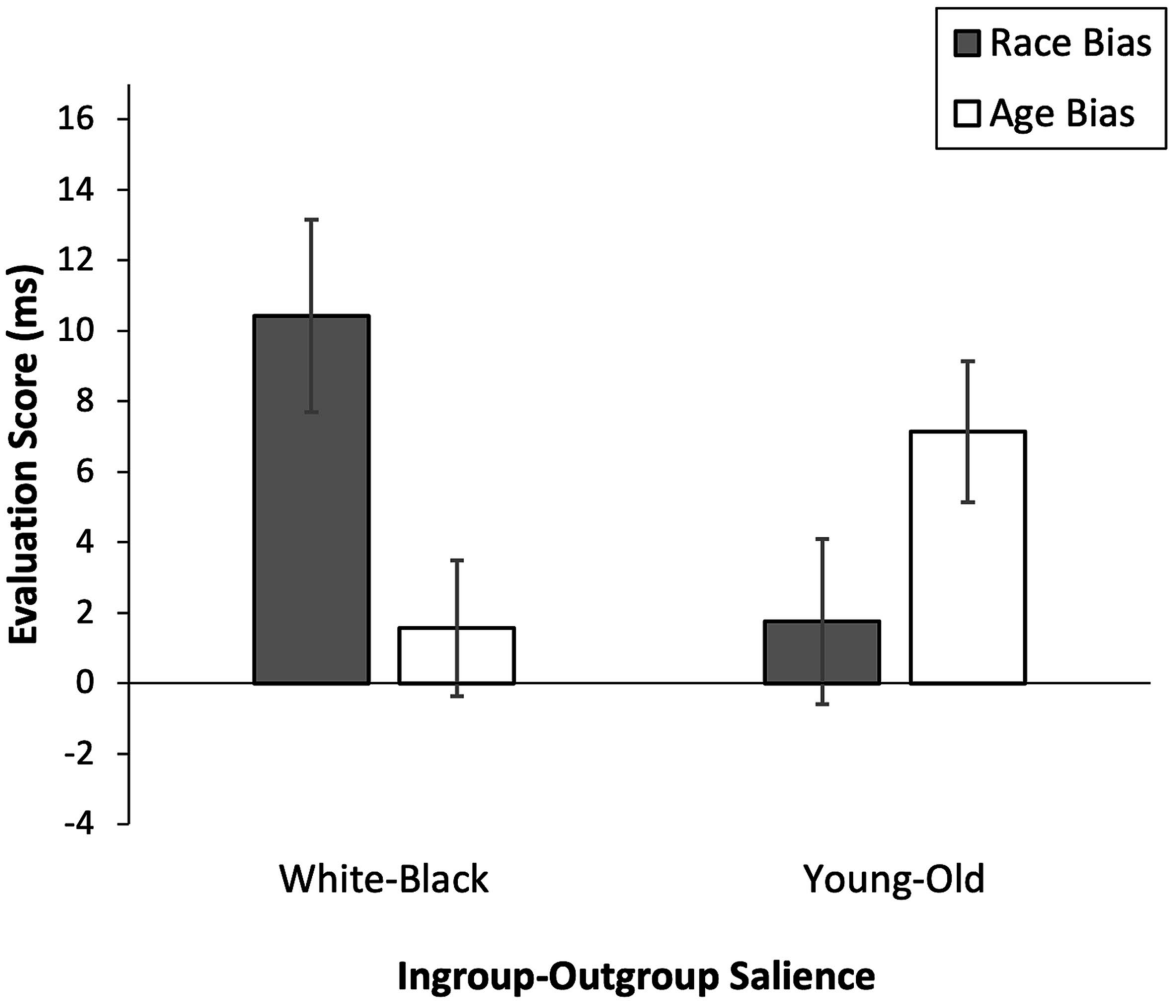
Automatic race bias and age bias scores as a function of Ingroup-Outgroup Salience (White-Black vs. Young-Old) in Experiment 1. Positive evaluation scores indicate preference for ingroup primes over outgroup primes. Higher scores indicate stronger preference. Error bars show standard errors of the mean.

We submitted the race and age bias scores to a 2 (ingroup-outgroup salience: White-Black vs. young-old) × 2 (evaluation score: race bias vs. age bias) mixed ANOVA for repeated measures with the first variable as a between-subjects factor. This analysis revealed the predicted two-way interaction of ingroup-outgroup salience and evaluation score, *F*(1, 69) = 11.48, *p* = .001, η*_p_*^2^ = .14, 90% CI [0.038, 0.267] (Figure 1). Simple comparisons (Bonferroni-corrected) showed that participants exhibited a higher race bias when White-Black was salient (*M* = 10.42, *SD* = 16.40) as compared to when young-old was salient (*M* = 1.75, *SD* = 13.84), *F*(1, 69) = 5.78, *p* = .019, η*_p_*^2^ = .08, 90% CI [0.007, 0.190]. Conversely, they exhibited a higher age bias when young-old was salient (*M* = 7.14, *SD* = 11.80) as compared to when White-Black was salient (*M* = 1.57, *SD* = 11.55), *F*(1, 69) = 4.04, *p* = .048, η*_p_*^2^ =.06, 90% CI [0.0002, 0.160]. Neither the main effect of ingroup-outgroup salience nor the main effect of evaluation score were significant, *F*s < 1, *p*s > .4, η*_p_*^2^ <.01, 90% CI [0, <0.08].

Finally, we tested whether automatic age bias and race bias scores differed significantly from zero. When White-Black was salient participants exhibited a significant automatic race bias, *t*(35) = 3.81, *p* < .001, 95% CI [4.88, 15.97], but no significant automatic age bias, *t*(35) = 0.81, *p* = .421, 95% CI  [−2.34, 5.48] (see Table 1 for effect sizes). Conversely, when young-old was salient participants exhibited a significant automatic age bias, *t*(34) = 3.58, *p* = .001, 95% CI  [3.08, 11.19], but no significant automatic race bias, *t*(34) = 0.75, *p* = .459, 95% CI  [−3.00, 6.51].

**Table 1:** Effect sizes of automatic race bias and age bias scores in experiments 1 and 2.

	Ingroup-outgroup salience with attribute description		Ingroup-outgroup salience without attribute description	
	White-Black	Young-Old	White-Black	Young-Old
Experiment 1				
*n*	36	35		
Race bias	0.64	0.13	-	-
	[0.273, 0.991]	[−0.207, 0.458]		
Age bias	0.14	0.61	-	-
	[−0.194, 0.463]	[0.240, 0.962]		
Experiment 2				
*n*	39	38	41	41
Race bias	0.43	0.11	0.68	−0.01
	[0.095, 0.751]	[−0.209, 0.429]	[0.340, 1.022]	[−0.317, 0.295]
Age bias	0.03	0.40	−0.03	0.31
	[−0.287, 0.341]	[0.064, 0.725]	[−0.331, 0.281]	[−0.002, 0.625]

Cohen’s d effect sizes of automatic race bias and age bias scores as a function of Ingroup-Outgroup Salience (White-Black vs. Young-Old) in Experiment 1, and as a function of Type of Manipulation (With vs. Without Attribute Description) in Experiment 2. 95% confidence intervals are shown in brackets. Cohen’s d for one-sample t-tests and confidence intervals were computed using the SPSS script CI-d-SPSS (http://core.ecu.edu/psyc/wuenschk/SPSS/CI-d-SPSS.zip).

#### 2.2.2 Self-reported evaluation

We calculated explicit race bias scores (i.e., scores of explicit preferences for White over Black targets), and explicit age bias scores (i.e., scores of explicit preferences for young over old targets) from the responses on the feeling thermometer items. To calculate explicit race bias scores, we subtracted the mean response to Black targets from the mean response to White targets. To calculate explicit age bias scores, we subtracted the mean response to old targets from the mean response to young targets.

A 2 (ingroup-outgroup salience: White-Black vs. young-old) × 2 (evaluation score: race bias vs. age bias) mixed ANOVA for repeated measures on explicit race and age bias scores with the first variable as a between-subjects factor revealed a significant main effect of evaluation score, *F*(1, 69) = 18.09, *p* < .001, η*_p_*^2^ = .21, 90% CI [0.080, 0.336], indicating that overall age bias scores (*M* = 0.14, *SD* = 0.67) were higher than race bias scores (*M* = −0.35, *SD* = 0.71). Note that race bias scores were negative, indicating that participants’ self-reported feelings toward Black people were warmer than their feelings toward White people. The main effect of evaluation score was further qualified by a significant interaction of ingroup-outgroup salience and evaluation score, *F*(1, 69) = 5.95, *p* = .017, η*_p_*^2^ =.08, 90% CI [0.008, 0.192]. Simple comparisons (Bonferroni-corrected) indicated that participants exhibited a lower age bias when young-old was salient (*M* = −0.02, *SD* = 0.57) as compared to when White-Black was salient (*M* = 0.30, *SD* = 0.72), *F*(1, 69) = 4.41, *p* = .039, η*_p_*^2^ = .06, 90% CI [0.002, 0.166]. Race bias scores did not differ significantly between conditions (White-Black salient: *M* = −0.47, *SD* = 0.45; young-old salient: *M* = −0.23, *SD* = 0.89), *F*(1, 69) = 2.00, *p* = .162, η*_p_*^2^ = .03, 90% CI [0, 0.117]. The main effect of ingroup-outgroup salience was not significant, *F*(1, 69) = 0.15, *p* = .699, η*_p_*^2^ = .002, 90% CI [0, 0.051].

#### 2.2.3 Social identification

To explore whether ingroup-outgroup salience affected identification with the salient ingroup, we submitted responses on the IIS to a 2 (ingroup-outgroup salience: White-Black vs. young-old) × 2 (identification group: White vs. young) mixed ANOVA for repeated measures with the first variable as a between-subjects factor. This analysis revealed a significant main effect of ingroup-outgroup salience, *F*(1, 69) = 5.39, *p* = .023, η*_p_*^2^ = .07, 90% CI [0.005, 0.183], suggesting that participants indicated higher identification with either group when White-Black was salient (identification with young people: *M* = 5.25, *SD* = 1.32; identification with White people: *M* = 5.17, *SD* = 1.38) as compared to when young-old was salient (identification with young people: *M* = 4.86, *SD* = 1.22; identification with White people: *M* = 4.40, *SD* = 1.33). Neither the main effect of identification group, *F*(1, 69) = 2.09, *p* = .153, η*_p_*^2^ =.03, 90% CI [0, 0.119], nor the interaction of ingroup-outgroup salience and identification group, *F*(1, 69) < 1, *p* = .321, η*_p_*^2^ = .01, 90% CI [0, 0.090], were significant.

### 2.3 Discussion

Experiment 1 shows that changes in ingroup-outgroup distinction salience moderate automatic intergroup bias. Automatic race bias was higher when White-Black was salient as compared to when young-old was salient. Conversely, automatic age bias was higher when young-old was salient as compared to when White-Black was salient. The findings support the assumption that ingroup-outgroup salience determines the ingroup/outgroup status of multiply categorizable targets, resulting in corresponding shifts in automatic intergroup evaluation.

The present results add to previous research in several ways. They show that the moderation of automatic evaluations of multiply categorizable targets is not limited to categorization manipulations directed toward the social targets presented in the automatic bias measure (Mitchell et al., 2003; Gawronski et al., 2010; Jones and Fazio, 2010; Todd et al., 2021). Instead, we directed participants’ attention to their own social identity as a White vs. young person, and how this social identity differs from the respective outgroup. This was sufficient to change automatic evaluations of multiply categorizable targets presented afterwards in a completely unrelated task. Compared to studies that employed a minimal group paradigm to manipulate social identity (Van Bavel and Cunningham, 2009), our findings show that the effects are not limited to a cooperative relationship to other-race ingroup targets, or to evaluative learning about the targets that are later presented in the evaluative bias measure. Instead, we observed changes in automatic evaluations of novel multiply categorizable targets that were completely irrelevant to our manipulation of ingroup-outgroup distinction salience.

Note that the observed pattern of results for self-reported evaluations diverged from the pattern of results for automatic evaluations. Ingroup-outgroup distinction salience affected explicit evaluations in the opposite direction than automatic evaluations: Participants exhibited less explicit age bias when young-old was salient as compared to when White-Black was salient. Conversely, explicit race bias scores were descriptively but not significantly lower when White-Black was salient as compared to when young-old was salient. Furthermore, race bias scores were overall negative, indicating a preference for Black over White people. Together, this pattern of results may suggest that self-reported responses were affected by social-desirability concerns. Moreover, making an ingroup-outgroup distinction salient may have specifically triggered correction processes with respect to the salient social category. Note, however, that this interpretation is post-hoc, and must therefore be treated with caution. Nevertheless, the discrepant findings on automatic vs. self-reported evaluations further stress the importance of automatic measures when investigating primary categories such as age and race.

One key feature of our ingroup-outgroup distinction salience manipulation is that participants describe themselves in terms of typical ingroup-attributes, which distinguish them from the respective outgroup. Given that people tend to think positively about themselves (Yamaguchi et al., 2007) and their ingroup (Volz et al., 2009), they may have listed mostly positive ingroup- and negative outgroup-attributes of the salient groups. Consequently, the valence of the activated group-associated attributes may have caused corresponding shifts in automatic evaluations (cf. Gramzow and Gaertner, 2005). We designed Experiment 2 to investigate whether the effects depend on this writing activity.

## 3 Experiment 2

### 3.1 Method

In Experiment 2, we manipulated the type of the ingroup-outgroup distinction salience manipulation. Following Ray et al. (2008), we told all participants that the data of White and Black (vs. young and old) participants would be compared. Then, participants indicated their group membership as White or Black (vs. young or old). Afterwards, half of the participants were asked to describe how they, as a White (vs. young) person, differed from Black (vs. old) people, replicating the procedure from Experiment 1. The other half of the participants continued to the next task without writing about group attributes. If we replicate the results from Experiment 1 in the latter condition, it appears unlikely that the effects are based on activating attributes of the salient ingroup and outgroup. The design was a 2 (ingroup-outgroup salience: White-Black vs. young-old) × 2 (type of manipulation: with vs. without attribute description) × 2 (evaluation score: race bias vs. age bias) mixed design with the latter variable manipulated within-subjects.

#### 3.1.1 Participants

Participants were 171 students recruited at a German university. They received a chocolate bar as compensation. As in Experiment 1, we excluded participants who were non-White (two participants) or aged 40 years or older (none). Seven participants were excluded because they did not select the option White or young, respectively, when asked about their group membership in the ingroup-outgroup salience task. One participant was excluded because she refused to list attributes that were typical of White people, stating that she differed from Black people in no respect. Two participants were excluded because they took part in the experiment the second time in a row. The age of the remaining 159 participants (94 females, 65 males) ranged from 16 to 31 years (*M* = 20.87, *SD* = 2.66). We determined the sample size based on Experiment 1 and conducted sensitivity power analyses with G*Power (Faul et al., 2007) separately for the Type of Manipulation conditions. With a sample size of *N* = 77 in the with-attribute-description condition and a correlation among the measures of *r* = 0.08, the minimum effect size of a between-within interaction effect we could detect with 80% power was *f* = 0.22. Given the observed effect size of η*_p_*^2^ = 0.07 (*f* = 0.28), power was sufficient in this condition. With a sample size of *N* = 82 in the without-attribute-description condition and a correlation among the measures of *r* = −0.002, the minimum effect size of a between-within interaction effect we could detect with 80% power was *f* = 0.22. Given the observed effect size of η*_p_*^2^ = 0.13 (*f* = 0.39), power was sufficient in this condition.

#### 3.1.2 Procedure and materials

##### 3.1.2.1 Manipulation of ingroup-outgroup distinction salience

After reading general instructions, half of the participants were told that the data of White and Black people would be compared and were asked to indicate their group membership as White or Black by selecting one of two boxes labeled “I am White” or “I am Black.” The other half of the participants were told that the data of young and old people would be compared and were asked to indicate their group membership as young or old by selecting one of two boxes labeled “I am young” or “I am old.”

After indicating their group membership, participants in the with-attribute-description condition additionally underwent the manipulation from Experiment 1. When White-Black was made salient, they were asked to describe how they, as a White person, differed from Black people. When young-old was made salient they were asked to describe how they, as a young person, differed from old people. Participants in the without-attribute-description condition did not complete this writing task but directly continued to the next task.

##### 3.1.2.2 Evaluative priming task (EPT)

Immediately after the manipulation, participants completed an EPT that was identical to the EPT in Experiment 1, with the exception that male participants were presented with photos of male persons as primes. These photos were 40 head-and-shoulder color photographs, 10 for each prime category (young White, old White, young Black, old Black), selected from the CAL/PAL Database (Minear and Park, 2004).

##### 3.1.2.3 Frequency of thoughts about group membership

To explore the mechanisms underlying the effects of ingroup-outgroup salience on automatic evaluation, we included a measure of frequency of thoughts about group memberships. Following the procedure from Wellen et al. (1998), participants indicated how often during the experiment their thoughts had been drawn to their group membership as a White person, and how often their thoughts had been drawn to their group membership as a young person. They responded on 7-point Likert scales ranging from 1 (*not at all*) to 7 (*all the time*). In addition, participants were asked to describe when during the experiment they thought of their group membership. The order of the group (young first, White second vs. White first, young second) was counterbalanced across participants.

##### 3.1.2.4 Self-stereotyping

Afterwards, participants completed a measure of self-stereotyping (Lun et al., 2009). Participants were presented with 48 traits, 12 of which were stereotypically associated with White people (Kawakami and Dovidio, 2001; Lun et al., 2009), 12 of which were stereotypically associated with Black people (Devine and Elliot, 1995; Lepore and Brown, 1997; Kawakami and Dovidio, 2001; Lun et al., 2009), 12 of which were stereotypically associated with young people (Chasteen et al., 2002), and 12 of which were stereotypically associated with old people (Hummert et al., 1995; Knox et al., 1995; Chasteen et al., 2002). Half of the traits were positive, and half of the traits were negative. Participants indicated how well each trait described themselves on a 7-point Likert scale ranging from 1 (*not at all*) to 7 (*very much*). All traits were presented in random order. Finally, participants answered demographic questions, were thanked and debriefed.

### 3.2 Results

#### 3.2.1 Automatic evaluation

As in Experiment 1, we discarded incorrect responses (4.8%) and responses that were faster than 150 ms (2 responses) or slower than 1,000 ms (3.0%). Means and standard deviations of the response latencies are presented in the Supplementary Table S1. Using the same procedure as in Experiment 1, we calculated automatic race bias scores and automatic age bias scores.

**Figure 2:**
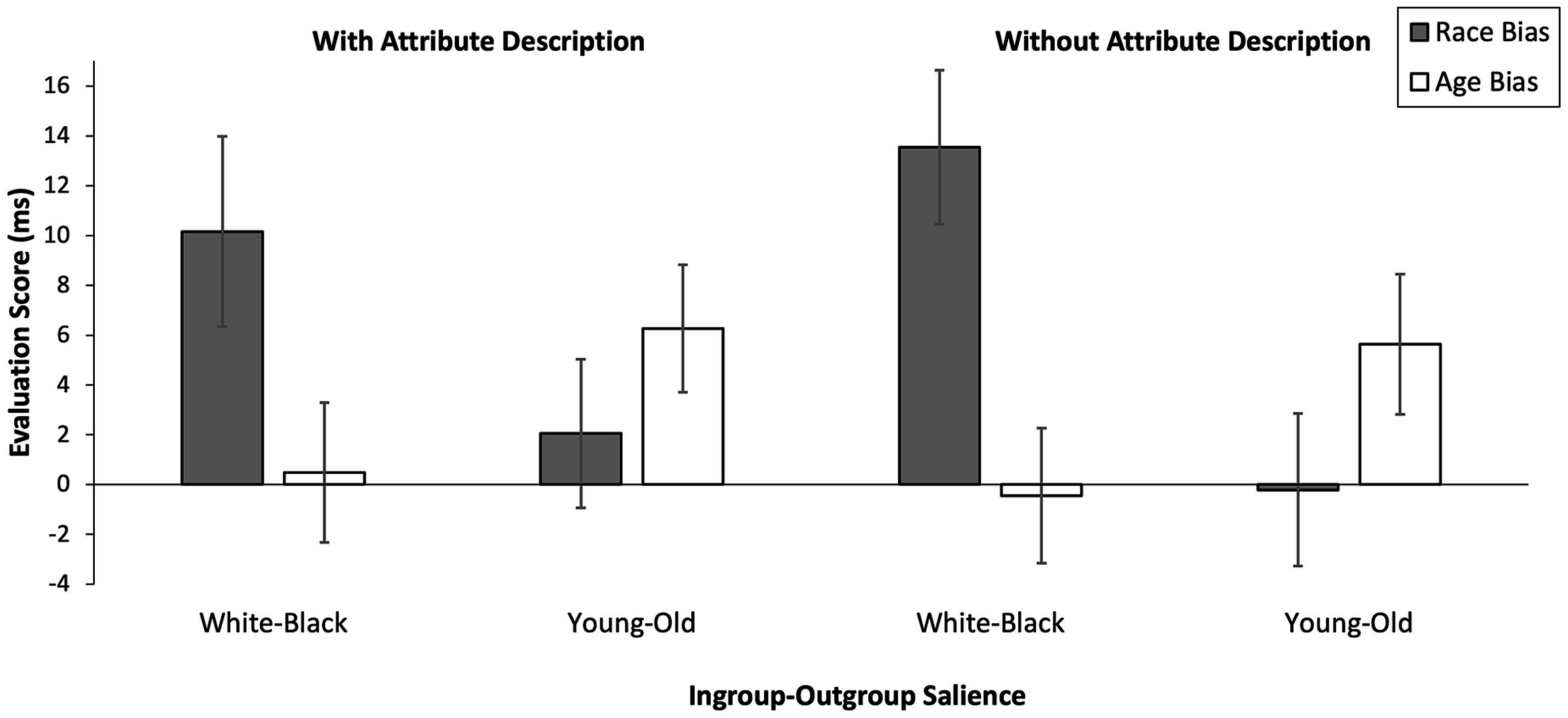
Automatic race bias and age bias scores as a function of Ingroup-Outgroup Salience (White-Black vs. Young-Old) and Type of Manipulation (With Attribute Description vs. Without Attribute Description). Positive evaluation scores indicate preference for ingroup primes over outgroup primes. Higher scores indicate stronger preference. Error bars show standard errors of the mean.

We submitted the race and age bias scores to a 2 (ingroup-outgroup salience: White-Black vs. young-old) × 2 (type of manipulation: with vs. without attribute description) × 2 (evaluation score: race bias vs. age bias) mixed ANOVA for repeated measures on evaluation scores with the first two variables as between-subjects factors (Figure 2). As expected, the two-way interaction of ingroup-outgroup salience and evaluation score was significant, *F*(1, 155) = 17.22, *p* < .001, η*_p_*^2^ =.10, 90% CI [0.037, 0.178]. Type of manipulation did not further moderate this interaction as indicated by a non-significant three-way interaction of ingroup-outgroup salience, type of manipulation, and evaluation score, *F*(1, 155) = 0.54, *p* = .466, η*_p_*^2^ = .003, 90% CI [0, 0.035]. All other main effects and interactions were not significant and irrelevant to our hypotheses, *F*s ≤ 2.81, *p*s ≥.096, η*_p_*^2^ ≤.02, 90% CI [0, ≤0.066]. Simple comparisons (Bonferroni-corrected) showed that participants exhibited a higher race bias when White-Black was salient (*M* = 11.90, *SD* = 21.79) as compared to when young-old was salient (*M* = 0.88, *SD* = 18.96), *F*(1, 155) = 11.28, *p* < .001, η*_p_*^2^ =.07, 90% CI [0.018, 0.139]. Conversely, participants exhibited a higher age bias when young-old was salient (*M* = 5.94, *SD* = 16.85) as compared to when White-Black was salient (*M* = 0.01, *SD* = 17.35), *F*(1, 155) = 4.72, *p* = .031, η*_p_*^2^ = .03, 90% CI [0.001, 0.085]. To further test whether the ingroup-outgroup salience × evaluation score interaction was significant in both Type of Manipulation conditions, we conducted separate two-way ANOVAs for the conditions.

##### 3.2.1.1 Salience manipulation with attribute description

A 2 (ingroup-outgroup salience: White-Black vs. young-old) × 2 (evaluation score: race bias vs. age bias) mixed ANOVA for repeated measures on evaluation scores with the first variable as a between-subjects factor revealed the predicted two-way interaction of ingroup-outgroup salience and evaluation score, *F*(1, 75) = 5.70, *p* = .019, η*_p_*^2^ =.07, 90% CI [0.006, 0.176]. The main effects were not significant, *F*s ≤ 0.88, *p*s ≥.351, η*_p_*^2^ ≤.01, 90% CI [0, ≤0.080]. The simple comparisons were not significant, but the mean differences were in the expected direction. Race bias scores were descriptively higher when White-Black was salient (*M* = 10.16, *SD* = 23.84) as compared to when young-old was salient (*M* = 2.05, *SD* = 18.46), *F*(1, 75) = 2.77, *p* = .100, η*_p_*^2^ =.04, 90% CI [0, 0.125]. Conversely, age bias scores were descriptively higher when young-old was salient (*M* = 6.26, *SD* = 15.77) as compared to when White-Black was salient (*M* = 0.48, *SD* = 17.52), *F*(1, 75) = 2.32, *p* = .132, η*_p_*^2^ = .03, 90% CI [0, 0.116].

As in Experiment 1, we tested whether evaluation scores differed significantly from zero (see Table 1 for effect sizes). When White-Black was salient participants exhibited a significant automatic race bias, *t*(38) = 2.66, *p* = .011, 95% CI  [2.43, 17.89], but no significant automatic age bias, *t*(38) = 0.17, *p* = .866, 95% CI  [−5.20, 6.15]. Conversely, when young-old was salient participants exhibited a significant automatic age bias, *t*(37) = 2.45, *p* =.019, 95% CI [1.08, 11.45], but no significant automatic race bias, *t*(37) = 0.68, *p* = .498, 95% CI  [−4.02, 8.12].

##### 3.2.1.2 Salience manipulation without attribute description

A 2 (ingroup-outgroup salience: White-Black vs. young-old) × 2 (evaluation score: race bias vs. age bias) mixed ANOVA for repeated measures on evaluation scores with the first variable as a between-subjects factor revealed the predicted two-way interaction of ingroup-outgroup salience and evaluation score, *F*(1, 80) = 12.23, *p* < .001, η*_p_*^2^ =.13, 90% CI [0.037, 0.248]. The main effects were not significant, *F*s ≤ 2.06, *p*s ≥.155, η*_p_*^2^ ≤.03, 90% CI [0, ≤0.104]. Simple comparisons (Bonferroni-corrected) showed that participants exhibited a higher race bias when White-Black was salient (M = 13.92, *SD* = 19.89) as compared to when young-old was salient (*M* = − 0.21, *SD* = 19.58), *F*(1, 80) = 10.01, *p* = .002, η*_p_*^2^ =.11, 90% CI [0.025, 0.223]. Age bias scores did not differ significantly but were descriptively higher when young-old was salient (*M* = 5.63, *SD* = 17.98) as compared to when White-Black was salient (*M* = −0.44, *SD* = 17.39), *F*(1, 80) = 2.42, *p* = .124, η*_p_*^2^ =.03, 90% CI [0, 0.111].

Furthermore, when White-Black was salient participants exhibited a significant automatic race bias, *t*(40) = 4.38, *p* < 0.001, 95% CI  [7.30, 19.80], but no significant automatic age bias, *t*(40) = 0.16, *p* = .872, 95% CI  [−5.93, 5.05] (see Table 1 for effect sizes). When young-old was salient participants exhibited neither a significant automatic age bias, *t*(40) = 2.01, *p* = .052, 95% CI  [−0.04, 11.31], nor a significant automatic race bias, *t*(40) = −0.07, *p* = .945, 95% CI  [−6.39, 5.97].

#### 3.2.2 Frequency of thoughts about group membership

We submitted responses on the 7-point Likert scales of thought frequency to a 2 (ingroup-outgroup salience: White-Black vs. young-old) × 2 (type of manipulation: with vs. without attribute listing) × 2 (ingroup: White vs. young) mixed ANOVA for repeated measures with the first two variables as between-subjects factors. This analysis revealed a significant two-way interaction of ingroup-outgroup salience and ingroup, *F*(1, 155) = 21.69, *p* < .001, η*_p_*^2^ = .12, 90% CI [0.053, 0.204], that was further qualified by a significant three-way interaction of type of manipulation, ingroup-outgroup salience, and ingroup, *F*(1, 155) = 6.37, *p* = .013, η*_p_*^2^ = .04, 90% CI [0.005, 0.100]. No other effects were significant, *F*s ≤ 3.44, *p*s ≥.066, η*_p_*^2^ ≤.02, 90% CI [0, ≤ 0.073].

Simple comparisons (Bonferroni-corrected) indicated that participants in the ingroup-outgroup salience with attribute description condition reported that they more frequently thought of being young when young-old was salient (*M* = 2.34, *SD* = 1.44) as compared to when White-Black was salient (*M* = 1.51, *SD* = 1.12), *F*(1, 155) = 8.91, *p* = .003, η*_p_*^2^ = .05, 90% CI [0.011, 0.121]. Conversely, they reported that they more frequently thought of being White when White-Black was salient (*M* = 2.64, *SD* = 1.61) as compared to when young-old was salient (*M* = 1.50, *SD* = 0.95), *F*(1, 155) = 12.01, *p* = .001, η*_p_*^2^ = .07, 90% CI [0.020, 0.144].

Participants in the ingroup-outgroup salience without attribute description condition also reported that they more frequently thought of being young when young-old was salient (*M* = 1.90, *SD* = 1.43) as compared to when White-Black was salient (*M* = 1.29, *SD* = 0.78), *F*(1, 155) = 5.13, *p* = .025, η*_p_*^2^ =.03, 90% CI [0.002, 0.089]. However, they did not more frequently think of being White, according to their self-report, when White-Black was salient (*M* = 1.95, *SD* = 1.58) as compared to when young-old was salient (*M* = 1.98, *SD* = 1.51), *F*(1, 155) < 0.01, *p* = .939, η*_p_*^2^ <.01, 90% CI [0, 0.002].

#### 3.2.3 Self-stereotyping

To simplify data analysis, we calculated race and age self-stereotyping scores. Specifically, we calculated self-stereotyping race scores by subtracting the mean of the stereotypical Black traits from the mean of the stereotypical White traits. Higher values indicate that participants ascribed more stereotypical White traits to themselves than stereotypical Black traits. To account for potential valence effects, we calculated separate scores for positive and negative traits, respectively. Furthermore, we calculated self-stereotyping age scores, by subtracting the mean of the stereotypical elderly traits from the mean of the stereotypical young traits. Higher values indicate that participants ascribed more stereotypical young traits to themselves than stereotypical elderly traits. Again, we calculated separate scores for positive and negative traits.

We submitted the self-stereotyping scores to a 2 (ingroup-outgroup salience: White-Black vs. young-old) × 2 (type of manipulation: with vs. without attribute listing) × 2 (self-stereotyping score: race vs. age) × 2 (trait valence: positive vs. negative) mixed ANOVA for repeated measures with the first two variables as between-subjects factors (see Supplementary Table S2 for descriptive statistics). This ANOVA revealed a significant main effect of self-stereotyping score, *F*(1, 155) = 16.76, *p* < .001, η*_p_*^2^ =.10, 90% CI [0.035, 0.175], a significant main effect of trait valence, *F*(1, 155) = 49.47, *p* < .001, η*_p_*^2^ = .24, 90% CI [0.149, 0.330], and a significant interaction of self-stereotyping score and trait valence, *F*(1, 155) = 127.58, *p* < .001, η*_p_*^2^ =.45, 90% CI [0.357, 0.527]. No other effects were significant, *F*s ≤ 3.29, *p*s ≥.072, η*_p_*^2^ ≤.02, 90% CI [0, ≤ 0.071].

### 3.3 Discussion

Experiment 2 replicates and extends the results from Experiment 1. Both types of ingroup-outgroup distinction salience manipulations (with and without attribute description) moderated automatic race and age bias. Making a particular ingroup-outgroup distinction salient by providing the information that data from the groups would be compared and by letting participants indicate their group membership was sufficient to change automatic group evaluations. Writing about typical group attributes did not increase the effect. If anything, the effect was descriptively smaller when participants were asked to describe themselves in terms of typical ingroup attributes (*f* = 0.28) as compared to when they merely indicated their group membership (*f* = 0.39). Thus, the results from Experiment 2 suggest that a simple shift in ingroup-outgroup distinction salience is sufficient to moderate automatic intergroup bias.

The results from the additional measures are in line with this view. Self-reported frequency of thoughts about group membership was affected by the ingroup-outgroup salience manipulation. Participants thought more frequently of the ingroup that was made salient. In contrast, self-stereotyping was not affected by the ingroup-outgroup salience manipulation. When discussing the results of Experiment 1, we had speculated that the attribute writing task may have let participants think of positive ingroup- and negative outgroup-attributes of the salient groups, which may have caused corresponding shifts in automatic evaluations. The findings from the self-stereotyping measure, however, do not confirm this view. Thus, it is more likely that a simple shift in ingroup-outgroup distinction salience caused the changes in automatic intergroup bias.

## 4 General discussion

The present research demonstrates that ingroup-outgroup distinction salience moderates automatic intergroup bias. Across two experiments and two different types of manipulations, automatic race bias was higher when Black-White was salient as compared to when young-old was salient. Conversely, automatic age bias was higher when young-old was salient as compared to when White-Black was salient. In Experiment 1, we manipulated ingroup-outgroup salience by letting participants describe how they as a White (vs. young) person differed from their respective outgroup. In Experiment 2, we informed participants that the data of White and Black people (vs. young and old people) would be compared and asked them to indicate their group membership as White or Black (vs. young or old). In a second condition, we asked participants to complete the same writing task as in Experiment 1. Regardless of whether participants engaged in this writing task or not, ingroup-outgroup salience moderated automatic intergroup bias.

Our research extends previous findings on the moderation of automatic evaluations of multiply categorizable targets (Mitchell et al., 2003; Gawronski et al., 2010; Jones and Fazio, 2010; Todd et al., 2021). Many studies directly or indirectly manipulated the categorization of the social stimuli that were presented during the automatic bias measure, for instance by asking participants to count Black and White faces (Gawronski et al., 2010; Jones and Fazio, 2010), or by varying distractor items presented in the automatic bias measure (Mitchell et al., 2003). Our studies show that the moderation of automatic evaluations of multiply categorizable individuals is not limited to manipulations that change the categorization of the individuals toward whom automatic evaluation is being measured. In other studies, participants completed a classification task before the automatic bias measure in which they repeatedly categorized multiply categorizable targets according to a particular dimension (Todd et al., 2021). Our studies show that the effects are not limited to practicing a categorization rule in a previous task. Other studies involved a learning phase during which participants categorized photos of White and Black individuals to an ingroup team and an outgroup team (Van Bavel and Cunningham, 2009). Our studies go beyond this research by showing that the effects are not limited to evaluative learning, or to framing the groups as teams which may enhance the connectedness to the ingroup members. Our research demonstrates that directing participants’ attention to their ingroup identity and the respective outgroup is sufficient to change automatic intergroup bias. Importantly, we demonstrate that our salience manipulation affected intergroup bias toward novel social stimuli that were presented after the salience manipulation during a task that was unrelated to the salience manipulation. These results suggest that shifts in salience of ingroup-outgroup distinctions may carry over from one context to another.

Future research may further examine the conditions under which the effects occur. For instance, one could investigate whether subliminal priming of the ingroup is sufficient to moderate automatic intergroup evaluations. A seminal study by Macrae et al. (1995) revealed that subliminal priming of social categories moderates stereotype accessibility. Thus, it may likely be that automatic intergroup evaluations can also be influenced by subliminal ingroup primes. Furthermore, future research may disentangle reduction and augmentation effects. Todd et al. (2021) observed that the magnitude of intergroup bias in a control condition was intermediate with that measured in the category salience conditions but did not differ significantly from either condition. Thus, salience manipulations may lead to both reduction and augmentation effects, but these effects appear to be very small, requiring huge sample sizes to provide sufficient statistical power. Importantly, the absence of a control condition in our design does not compromise our conclusion that ingroup-outgroup distinction salience moderates automatic intergroup bias. Finally, future research may investigate the role of ingroup identification. Based on research from Roth and Steffens (2014), one would predict that changes in automatic intergroup attitudes will be larger when identification with the salient ingroup is higher (see also Roth et al., 2018).

In sum, the present research suggests that a simple shift in ingroup-outgroup distinction salience moderates automatic age and race bias toward multiply categorizable targets. As such, our findings contribute to a better understanding of the determinants of automatic intergroup bias.

## Data availability statement

The datasets presented in this study can be found in online repositories. The names of the repository/repositories and accession number(s) can be found at: https://osf.io/7g8va/.

## Ethics statement

Ethical approval was not required for the studies involving humans because participants were not expected to take risks, because the studies did not involve a high level of physical or emotional stress, and because participants were fully debriefed (see the principles of the German Science Foundation https://www.dfg.de/en/research_funding/faq/faq_humanities_social_science/index.html). The studies were conducted in accordance with the local legislation and institutional requirements. The participants provided their written informed consent to participate in this study.

## Author contributions

SH: Conceptualization, Data curation, Formal analysis, Methodology, Visualization, Writing – original draft, Writing – review & editing, Investigation, Software. RR: Conceptualization, Data curation, Formal analysis, Methodology, Visualization, Writing – original draft, Writing – review & editing, Supervision.

## Footnotes

^1^ https://osf.io/7g8va/

^2^ http://core.ecu.edu/psyc/wuenschk/SPSS/CI-R2-SPSS.zip

^3^ http://daniellakens.blogspot.com/2014/06/calculating-confidence-intervals-for.html
