# Supplementary material for: Determinants of automatic age and race bias: ingroup-outgroup distinction salience moderates automatic evaluations of social groups
Source: Front Psychol. 2024 Mar 18;15:1328775. doi: 10.3389/fpsyg.2024.1328775 (PMC10982430; doi:10.3389/fpsyg.2024.1328775)
Supplement: Supplementary file 1 [file Table_1.pdf]

Supplementary Material

Heitmann & Reichardt

Determinants of Automatic Age and Race Bias: Ingroup-Outgroup Distinction Salience Moderates Automatic Evaluations of Social Groups

Table S1. Means and standard deviations of response latencies (in ms) in the Evaluative Priming Task

Ingroup-Outgroup Salience	Target	Young White Prime	Old White Prime	Young Black Prime	Old Black Prime
Experiment 1: Salience Manipulation with Attribute Description					
White-Black	Positive	565 (49)	562 (46)	578 (48)	572 (51)
	Negative	595 (49)	584 (47)	581 (48)	578 (41)
Young-Old	Positive	587 (49)	592 (50)	589 (51)	599 (46)
	Negative	603 (52)	593 (56)	601 (55)	597 (53)
Experiment 2: Salience Manipulation with Attribute Description					
White-Black	Positive	579 (56)	582 (54)	597 (64)	592 (60)
	Negative	593 (60)	588 (57)	584 (57)	584 (60)
Young-Old	Positive	562 (61)	574 (58)	567 (63)	577 (56)
	Negative	577 (60)	575 (57)	577 (62)	576 (63)
Experiment 2: Salience Manipulation without Attribute Description					
White-Black	Positive	582 (61)	584 (63)	598 (66)	591 (64)
	Negative	598 (61)	598 (62)	584 (58)	581 (58)
Young-Old	Positive	555 (56)	565 (65)	563 (58)	567 (61)
	Negative	566 (55)	555 (54)	565 (55)	567 (64)

Note: Mean response latencies (in ms) as a function of Prime (young White vs. old White vs. young Black vs. old Black), Target Valence (positive vs. negative), and Ingroup-Outgroup Salience (White-Black vs. young-old) in Experiment 1, and as a function of Type of Manipulation (with vs. without attribute description) in Experiment 2. Standard deviations are printed in parentheses.
