# Supplementary material for: Determinants of automatic age and race bias: ingroup-outgroup distinction salience moderates automatic evaluations of social groups
Source: Front Psychol. 2024 Mar 18;15:1328775. doi: 10.3389/fpsyg.2024.1328775 (PMC10982430; doi:10.3389/fpsyg.2024.1328775)
Supplement: Supplementary file 2 [file Table_2.pdf]

Supplementary Material

Heitmann & Reichardt

Determinants of Automatic Age and Race Bias: Ingroup-Outgroup Distinction Salience Moderates Automatic Evaluations of Social Groups

Table S2. Means and standard deviations of self-stereotyping scores

	Ingroup-Outgroup Salience with Attribute Description		Ingroup-Outgroup Salience without Attribute Description	
	White-Black	Young-Old	White-Black	Young-Old
Race Self-Stereotyping				
Positive Traits	0.36 (0.80)	0.06 (0.85)	0.28 (0.79)	0.21 (0.81)
Negative Traits	0.53 (0.74)	0.52 (0.81)	0.41 (0.72)	0.39 (0.74)
Age Self-Stereotyping				
Positive Traits	0.34 (0.73)	0.58 (0.77)	0.44 (0.89)	0.67 (0.82)
Negative Traits	-0.51 (0.96)	-0.32 (0.93)	-0.58 (0.89)	-0.54 (0.92)

Note: Mean self-stereotyping scores as a function of Ingroup-Outgroup Salience (White-Black vs. young-old), Type of Manipulation (with vs. without attribute description), Self-Stereotyping Score (race vs. age), and Trait Valence (positive vs. negative) in Experiment 1. Self-stereotyping scores were calculated by subtracting the mean value of self-ascription of outgroup traits from the mean value of self-ascription of ingroup traits, separately for positive and negative race and age traits. Higher values indicate stronger ascription of ingroup- relative to outgroup-traits to the self. Standard deviations are printed in parentheses.
